# Effect of Auditory Discrimination Therapy on Attentional Processes of Tinnitus Patients

**DOI:** 10.3390/s22030937

**Published:** 2022-01-26

**Authors:** Ingrid G. Rodríguez-León, Luz María Alonso-Valerdi, Ricardo A. Salido-Ruiz, Israel Román-Godínez, David I. Ibarra-Zarate, Sulema Torres-Ramos

**Affiliations:** 1 Division of Cyber-Human Interaction Technologies, University of Guadalajara (UdG), Guadalajara 44100, Jalisco, Mexico; ingrid.rleon@alumnos.udg.mx (I.G.R.-L.); ricardo.salido@academicos.udg.mx (R.A.S.-R.); israel.roman@academicos.udg.mx (I.R.-G.); 2 Tecnologico de Monterrey, Escuela de Ingenieria y Ciencias, Monterrey 64849, Nuevo Leon, Mexico; lm.aloval@tec.mx (L.M.A.-V.); david.ibarra@tec.mx (D.I.I.-Z.)

**Keywords:** tinnitus, auditory discrimination therapy, EEG evaluation, event-related synchronization, event-related desynchronization, convolutional neural network

## Abstract

Tinnitus is an auditory condition that causes humans to hear a sound anytime, anywhere. Chronic and refractory tinnitus is caused by an over synchronization of neurons. Sound has been applied as an alternative treatment to resynchronize neuronal activity. To date, various acoustic therapies have been proposed to treat tinnitus. However, the effect is not yet well understood. Therefore, the objective of this study is to establish an objective methodology using electroencephalography (EEG) signals to measure changes in attentional processes in patients with tinnitus treated with auditory discrimination therapy (ADT). To this aim, first, event-related (de-) synchronization (ERD/ERS) responses were mapped to extract the levels of synchronization related to the auditory recognition event. Second, the deep representations of the scalograms were extracted using a previously trained Convolutional Neural Network (CNN) architecture (MobileNet v2). Third, the deep spectrum features corresponding to the study datasets were analyzed to investigate performance in terms of attention and memory changes. The results proved strong evidence of the feasibility of ADT to treat tinnitus, which is possibly due to attentional redirection.

## 1. Introduction

Tinnitus is the perception of sound in the absence of an external source [1]. It affects between 5 and 15% of the world population [2]. Tinnitus is caused by exposure to loud noise, fever, ototoxicity, or a transient disturbance in the middle ear [1]. Tinnitus can be perceived by people of all ages, either those with normal hearing or those with hearing loss [3]. Lenhardt classified tinnitus into objective and subjective [4]. Objective tinnitus is associated with peripheral vascular abnormalities detectable by stethoscopic inspection, whereas subjective tinnitus is determined as an acoustic perception merely experienced by the patient [5]. The tinnitus of interest for the present investigation is the subjective one.

Subjective tinnitus can become chronic and refractory, and it may be caused by the over synchronization of neurons, which affects cognitive, attentional, emotional, and even motor processes [1]. Cognitive impairment has been frequently reported in patients with tinnitus over the last few years [6]. Particularly, working memory and attentional processes that are affected include deficits in (1) executive control of attention [7], (2) attentional changes [6], and (3) selective and divided attention [8]. Furthermore, tinnitus differs across patients in its perceptual characteristics (e.g., frequency and intensity), in its time course (constant, fluctuating, and intermittent), response to interventions (e.g., masking sounds and somatic maneuvers), etiologic factors, and comorbidities [9]. This heterogeneity of tinnitus is reflected by a substantial variability in tinnitus pathophysiology [10], which causes a high variability in the treatment outcome. Therefore, a major challenge in clinical tinnitus research is the identification of relevant criteria for subtyping patients [11,12].

The attentional neurophysiological mechanisms altered by the presence of tinnitus can be recorded over the human scalp using the electroencephalography (EEG) technique [13]. EEG allows monitoring neural oscillations and ongoing electrical activity, which is made up of several simultaneous oscillations at different frequencies [14,15,16]. Neural oscillations have traditionally been studied based on event-related experiments, where event-related potentials and (de-) synchronization levels have been estimated [5]. Specifically, event-related neural oscillatory responses at different frequency bands reflect different stages of neural information processing [14,15,17]. Event-related oscillations are typically studied as (1) event-related desynchronization (ERD), which refers to the phasic relative power decrease of a certain frequency band, and (2) event-related synchronization (ERS), which implies a relative power increase. As the term indicates, both ERD and ERS are neural patterns occurring in relation to emotional, cognitive, motor, sensory, and/or perceptual events [18,19,20]. In tinnitus patients, power changes in various frequency bands reflects changes in neural synchrony [5]. The levels of synchronization related to auditory stimuli are carried out here to evaluate the effect of auditory discrimination therapy (ADT).

It is well established that sound brings about physiological, cognitive, and psychological changes, which is why sound-based therapies have become seven of the twenty-five most widely used treatments for tinnitus according to [12]. ADT is an acoustic therapy based on the oddball paradigm principle. This therapy is designed to reduce attention toward tinnitus, thereby reducing its perception [21]. The oddball paradigm consists of a pair of stimuli: standard and deviant pulses, which are randomly presented. The patient must identify deviant (40%) from standard (60%) pulses. This therapy intends to redirect the patient attention toward other sensorial events different from tinnitus so as to reduce its perception. It requires the attention of the patient on the therapy by presenting a composed sound of standard and deviant pulses in a random way. The patient must identify which type of pulse is presented, either standard or deviant. The standard pulse is the same tone that the tinnitus is, and the deviant pulse is 10% more than the standard one. Auditory discrimination has shown an improvement in tinnitus symptoms attributed to the rehabilitation of auditory processing frequencies of the auditory cortex damaged due to tinnitus [22] and prevention of auditory cortex reorganization [23]. Training at tones that differed from the dominant tinnitus pitch is beneficial due to the effect of lateral inhibition. Furthermore, stimulating specific frequency regions close to but not within the tinnitus frequency region will likely promote or strengthen lateral inhibitory activity, thus disrupting the pathological synchronous activity of the tinnitus-generating region [24]. There are currently several areas of opportunity suggested by the scientific community to study [25]. A distinctive niche refers to finding objective measures to evaluate the effect of treatments in patients with tinnitus, since there are conventional clinical protocols based on a trial-and-error procedure, and there is no formal and adequate follow-up of the treatment. At present, the most used way to evaluate acoustic therapies is through subjective methods such as the visual analogue scale and ad hoc questionnaires [3]. For instance, [26] evaluated the effectiveness of using sound generators with individual adjustments to relieve tinnitus in patients unresponsive to previous treatments and according to the Tinnitus Handicap Inventory (THI) test. The authors found improvement in quality of life, with good response to sound therapy. Not only subjective but also objective evaluation has been recently undertaken. The investigation presented by [27] compared sound therapies based on music, retraining, neuromodulation (e.g., ADT), and binaural sounds using neuro-audiology assessments and psychological evaluations. The first assessment revealed that the whole frequency structure of the neural networks showed a higher level of activeness in tinnitus sufferers than in control individuals. According to the psychological evaluation, the retraining treatment was the most effective sound-based therapy to reduce tinnitus perception and to release stress and anxiety after 60 days of treatment. Nonetheless, binaural sounds and ADT produced very similar effects. Furthermore, ADT showed to exert less side effects. Secondly, [28] evaluated the feasibility of Binaural Sound Therapy (BST) for tinnitus treatment by comparing its effect with Music Therapy (MT) effect. According to the THI questionnaire outcomes, BST reduced tinnitus perception. On the other hand, slightly major neural synchronicity over the right frontal lobe was reflected after two-month treatment.

In the light of the above discussion, the present work aims to establish a methodology based on EEG analysis to evaluate objectively the effectiveness of ADT to redirect the attention of patients with tinnitus. For this purpose, the database “Acoustic therapies for tinnitus treatment: An EEG database” [29] was used. From the database, only control and ADT groups were selected. Afterwards, ERD and ERS responses were mapped for two study cases: (1) before and (2) after applying the ADT. For ERD–ERS maps, Continuous Wavelet Transform (CWT) related to auditory material recognition was computed. Thereafter, deep representations from the resulting scalograms images using pre-trained Convolutional Neural Networks (CNNs) were extracted. Finally, deep spectrum features were analyzed to investigate the performance in terms of cognitive changes, specifically those related to attention and memory. The foregoing may provide solid evidence of the feasibility of ADT to treat subjective, chronic, and refractory tinnitus. The conduction of the investigation is described below.

## 2. Materials and Methods

**Figure 1:**
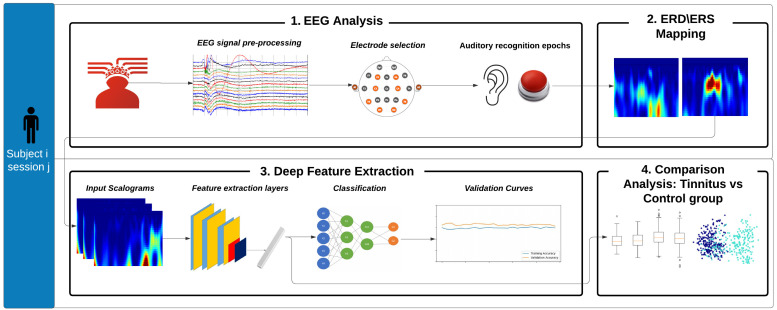
Four-step based methodology followed for the current research study: (1) EEG Analysis, (2) ERD/ERS Mapping, (3) Deep Feature Extraction, and (4) Comparison Analysis: Tinnitus vs. Control group.

The methodology for this work was undertaken into four steps: (1) to analyze and select the EEG signals of interest from the aforementioned database, (2) to estimate the ERD/ERS maps based on CWT, (3) to extract deep features based on CNN, and (4) to analyze statistically data based on centroids and Euclidean distances. This methodology is shown in Figure 1 and described in detailed in the following paragraphs.

### 2.1. EEG Database

The database for this research is available at Mendeley Data under the title “Acoustic therapies for tinnitus treatment: An EEG database” [29]. This database was created by following a protocol formerly approved by the Ethical Committee of the National School of Medicine of the Tecnologico de Monterrey, described, published, and registered under the trial number: ISRCTN14553550.

From the cohort, two groups were selected: tinnitus patients treated with ADT and controls. There were eleven participants per group. Both groups were treated for 8 weeks and were instructed to use the sound-based therapy for one hour every day at any time of the day. Note that controls were acoustically stimulated with relaxing music. In both cases, the sound therapy was monitored by psychometric and electroencephalographic evaluations before and after the 8-week treatment. For the EEG monitoring, four auditory stimulation conditions were found: (1) 3 min at resting state, (2) 3 min at listening to the corresponding therapy, (3) 2.5 min at listening to intermittent stimuli, and (4) 5 min at listening to everyday soundscapes where individuals had to identify 5 different sounds. The last case was the only one analyzed for this research. As this research aimed to evaluate objectively the effectiveness of ADT to redirect the patient’s attention, the EEG analysis of tinnitus patients when recognizing everyday sounds (e.g., mobile ring, car horn) at common soundscapes could reveal whether the tinnitus attention had been reduced, and they were able to identify those sounds.

**Figure 2:**
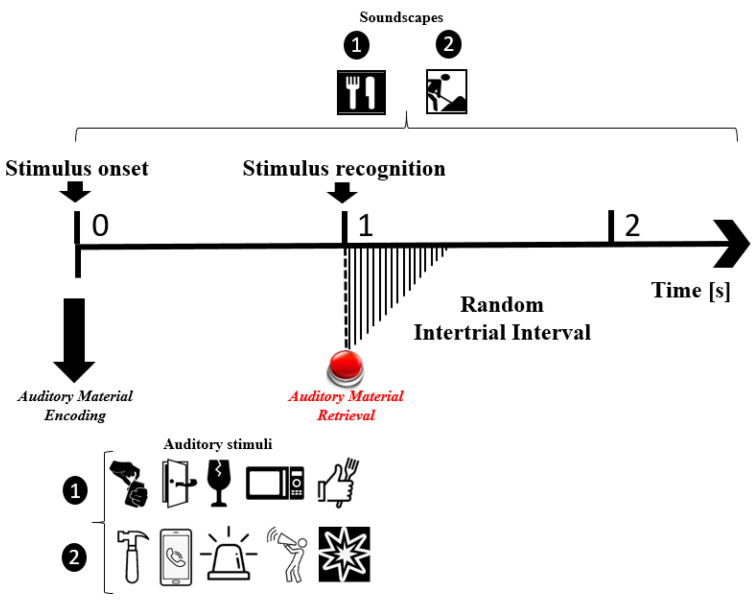
Timing protocol for EEG data in use. Each trial was around 60 min long. In each trial, participants listened to a soundscape and identified five randomly played auditory stimuli by pressing a button on the keyboard. There were two types of induced events: (1) auditory material encoding and (2) auditory material retrieval.

Two different soundscapes were played, while five associated auditory stimuli were randomly played. Whenever participants identified auditory stimuli, they pressed a keyboard button. The soundscapes and their related auditory stimuli to be identified for each monitoring session were: (1) *construction in progress*: (i) human sound (yelling), (ii) police siren, (iii) mobile dialing, (iv) bang, and (v) hit; and (2) *restaurant*: (i) human sound (tasting food), (ii) microwave sound, (iii) glass breaking, (iv) door closing, and (v) soda can being opened. All the stimuli lasted 1 s and were repeated 50 times at a random rate. Participants kept their eyes closed during the stimulation. Every monitoring session was around 60 min long [3]. The experimental timing protocol is illustrated in Figure 2.

To record the EEG data, a g.USBamp amplifier was used, which was configured as stated in Table 1. Furthermore, clinical (level of hearing loss and frequency, intensity, and laterality of tinnitus) and demographic (gender, age) characteristics from the cohort selected were registered.

### 2.2. EEG Signal Pre-Processing

The EEG signals were pre-processed as follows. Firstly, the low-frequency components were eliminated by applying a Butterworth-type Band Pass digital filter with order 6 of zero phase, and with cutoff frequencies between 0.1 and 30 Hz. Secondly, channels were removed according to the criteria reported in [30]: flat for more than 5 s, maximum acceptable high-frequency noise standard deviation of 4, minimum acceptable correlation with nearby channels of 0.8. Thirdly, Artifact Subspace Reconstruction (ASR) bad burst correction was performed in order to remove bad data periods with transient or large-amplitude artifacts that exceeded 20 times the standard deviation of the calibrated data [30]. Fourthly, Independent Component Analysis (ICA) was applied with RunICA function. Finally, the independent components (ICs) distinguished as non-brain sources were rejected by the ICLabel classifier. The probability range for components flagged for rejection was set between 0.6 and 1. There were five non-brain source categories: (1) muscular, (2) ocular, and (3) electrocardiographic artifacts, (4) line noise, and (5) channel noise.

Due to the previous pre-processing stage alongside with some missing material recognition responses in the initial monitoring session, there was a significant loss of auditory material retrieval events; therefore, the sample of interest had to be reduced to 5 tinnitus patients composed of four adults aged 30–59 years old and one elderly aged 60–85 years old: 3 males and 2 females.

Table A1 (located in Appendix A) shows up the rejected channels, the percentage of bad data periods with transient or large-amplitude artifacts, and the independent components distinguished as non-brain sources.

### 2.3. ERD/ERS Maps

To begin this process, EEG signals over the frontal lobe and middle line (Fz) were carried out to monitor the ADT effect on tinnitus sufferers. Channel Fz was selected to analyze EEG information, since it is the recording site for clinical diagnosis of tinnitus.

Secondly, the epochs were extracted 500 ms before and 1 s after the keyboard button press; i.e., the recognition of the familiar sound played randomly during the everyday soundscape (Figure 2). This event refers to the auditory material retrieval. A negative window was proposed as a reference to measure changes in potential prior to the event whilst the positive window is aligned with the timing protocol corresponding to the time of appearance of ERD/ERS responses associated with the auditory memory and attentional mechanisms involved [31].

Thirdly, the CWT was the time-frequency analysis applied to each of 50 epochs per stimulus (5 stimuli in total). Wavelet of the Complex Gaussian family (Equation (1)) was selected, since they are based on complex-valued sinusoids constituting an analytic signal, possessing the shift invariance property. The sampling frequency was 256 Hz. The frequency range oscillated between 0.1 and 30 Hz.

(1) $$f(x)=C_{p}e^{-ix}e^{-x^{2}}$$

The integer *p* is the parameter of this family built from the complex Gaussian function. *C_p_* is such that $∥f^{p}{∥}^{2}=1$ where *f^p^* is the *p^th^* derivative of *f*.

Fourthly, the baseline correction (*BC*) was carried out using the subtraction method based on Equation (2).

(2) $$BC=\left(P\left(t,f\right)-\bar{R}\left(f\right)\right)$$

where *P*(*t*,*f*) is the power value given a time-frequency point subtracted by the average value of the baseline values from −400 to −100 ms at each frequency range prior to the appearance of an auditory recognition event [32].

Finally, the coefficient matrices resulting from the CWT per epoch were averaged, and the absolute value was carried out to obtain only real estimations. CWT scalograms were plotted as a function of time windows from −500 ms to 1 s and a frequency ranging from 0.1 to 30 Hz, for the purpose of representing the auditory synchronization and desynchronization activity over the Fz area before and after the ADT-based procedure.

### 2.4. Deep Feature Extraction

The CNN is often used in disease detection and classification [33,34]. Nonetheless, in this paper, it was executed with the aim of extracting a distributed vector representation of the scalograms images resulted from training a model to classify tinnitus from control patients. From now on, such vector representations will be known as *deep spectrum features*. The premise with such deep spectrum features is that images from tinnitus patients result in vector representations that are closer among them and, at the same time, distant from vector representations corresponding to control participants. The CNN utilized was the MobileNet V2, which is based on a streamlined architecture that uses depth-wise separable convolutions, a form of factorized convolutions, with the aim to build lightweight deep neural networks. MobileNet uses 3 × 3 depth wise separable convolutions, which uses between 8 and 9 times less computation, and it is extremely efficient relative to standard convolutions. Furthermore, the model has the effect of drastically reducing model size and computational cost [35]. This feature helps face the high computing capability and the large memory requirements characterized in a CNN method [33]. The pre-trained CNN was transferred to our recognition of auditory material task for extracting the deep spectrum features from the scalogram images carried out in the previous section.

The dataset used was 2468 scalogram images, divided into four classes, tinnitus patients before (801 images) and after (667 images) the treatment and control subjects before (500 images) and after (500 images) the treatment. There is a significantly larger number of tinnitus samples compared to the control ones (approximately 59% against 41%, respectively).

The pixel values in the images were into the range [0, 255]. So, as part of the model expectation, the pre-processing method included with the CNN model was executed to rescale the pixel values in [−1, 1]. Furthermore, the scalograms were resized from 1200 × 900 to 160 × 160.

To start with, the base model from the MobileNet-V2, which is pre-trained on the ImageNet dataset model, was executed to classify between controls and tinnitus patients before the corresponding sound-based treatment.

Secondly, the feature extractor converted each 160 × 160 × 3 image into a 5 × 5 × 1280 block of features. Hence, a classifier was added on top of it so the top-level classifier can be trained accordingly.

Thirdly, in order to generate predictions from the block of features, a GlobalAveragePooling2D layer was used to average over the spatial 5 × 5 spatial locations with the aim to convert the features to a single 1280-element vector per image. In addition, a Dense layer was applied to convert these features into a single prediction per image. Positive numbers predicted class 1 (Control participants), and negative numbers predicted class 0 (Tinnitus patients). There were 1.2K trainable parameters in the Dense layer, which were divided in 2 variable objects: the weights and biases.

**Figure 3:**
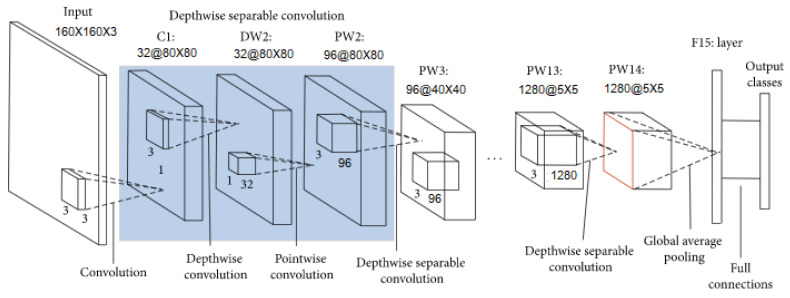
MobileNet-V2 architecture.

Fourthly, the model was compiled. An Adam optimizer was used with a learning rate of 1 × 10^−4^, dropout value of 0.2, and a batch size of 32. The architecture of the model executed is shown in Figure 3. An exhaustive search was executed to find optimal learning, epochs, batch size rate, and dropout values hyper parameters in the classifier block; learning rates from 1 × 10^−3^ to 1 × 10^−6^, dropout values from 0.1 to 0.5, epochs from 15 to 100, and batch size from 25 to 45 were explored.

Fifthly, the MobileNet-V2 base model was trained by using 25 epochs. Learning curves of the training and validation accuracies were plotted (Figure A1 located in Appendix A), getting 69% accuracy on the validation set. An 80/20 validation was applied: 80% of data was used for model construction, and 20% of the data was used for model validation. The validation metrics were evaluated after the corresponding epochs.

Finally, the convolutional base, pre-loaded with weights trained on ImageNet without the classification layers, was applied for the feature extraction of scalogram images related to the auditory material recognition task carried out from tinnitus patients and controls during the two monitoring sessions: before and after the corresponding sound-based treatment.

### 2.5. Comparison Analysis: Tinnitus vs. Control Group

Once deep spectrum features were extracted per scalogram, in order to analyze tinnitus and control groups, a statistical evaluation was performed to acquire the significant differences among all the study datasets. Furthermore, an estimator was calculated to evaluate the effect of the sound-based therapy, and finally, centroids and distances were obtained to measure the closeness between the instances of the tinnitus group and control group.

#### 2.5.1. Statistical Evaluation

The statistical analyses were conducted separately for each dataset: tinnitus patients and controls before and after the treatment considering the recognition of auditory material.

The Lilliefors test was used to assess data distribution between-tinnitus subjects, within-tinnitus subjects, and within-control subjects before and after the sound-based treatments. After achieving a normal distribution, the statistical significance of any differences among the groups stated in Table 2 was evaluated with the Student’s *t*-test. *p*-values were stated at 5% for both statistical processes. *p*-values greater than 0.05 will represent a statistically significant relationship in ERD/ERS responses between the indicated study data sets, whilst *p*-values less than 0.05 will show significant differences. Significant relationship responses between the tinnitus group after the sound-based treatment versus control group could help point out whether ADT was a reliable treatment. Additionally, box plots were created.

#### 2.5.2. The Differences in Differences (DID) Estimator

The DID estimator was estimated to analyze the differential effect of the sound-based treatment on the tinnitus group versus the control group in both experimental designs: between subjects and within subjects. The DID model is based on Equation (3).

(3) $$Y=\beta_{0}+\beta_{1}Time+\beta_{2}Intervention+\beta_{3}\left(Time\cdot Intervention\right)+\varepsilon$$

where *β*_0_ is the baseline average, *β*_1_ is the time trend in the control group, *β*_2_ is the difference between two groups pre-intervention, and *β*_3_ is the difference in changes over time.

DID is a quasi-experimental design that makes use of longitudinal data from treatment and control groups to estimate a causal effect of a specific intervention or treatment by comparing the changes in outcomes over time. DID requires data from pre-/post-intervention, such as cohort or repeated cross-sectional data. The approach gets rid of biases in post-intervention period comparisons between the treatment and control group and from comparisons over time in the treatment group [36].

#### 2.5.3. Centroid and Distance Measures

Firstly, there were calculated centroid values based on the mean values of the coordinates of all the data instances from control and tinnitus groups before and after the treatment (Equation (4)).


(4) $$C_{i}=\frac{1}{p}\overset{p}{\underset{j=1}{\sum }}x_{i}^{j}$$

$x^{u}$ is the *u*-th deep spectrum feature vector where $x^{u}\in R^{1280}$, u∈{1,2,…,p} (*p* is the number of scalograms for a given group). Additionally, i∈{1,2,…,1280} where *i* is the *i*-th component of the vector *x*.

Secondly, Euclidian distance was calculated between each data instance and the corresponding centroids (Equation (5)). Media (Equation (6)) and standard deviations (Equation (7)) were reported. By applying the present criteria, it was possible to measure the closeness between the instances of the tinnitus group after receiving the therapy with respect to the control centroids. Analysis based on centroids and distances offered a novel multidimensional approach for identifying tinnitus groups already treated that exhibited similarities in ERD/ERS responses compared with control groups. If the mean Euclidian distance between the instances of the tinnitus group after treatment and the centroids of the control group is shorter than the corresponding between the instances of the tinnitus group before treatment and the centroids of the control group, this could indicate the existence of neural similarities, which could support the effectiveness of treatment in some scenarios.

(5) $$D=\sqrt{{\left(x_{1}^{u}-C_{1}^{k}\right)}^{2}+{\left(x_{2}^{u}-C_{2}^{k}\right)}^{2}+\ldots +{\left(x_{1280}^{u}-C_{1280}^{k}\right)}^{2}}$$

where $x^{u}$ is a deep spectrum feature vector and $C^{k}$ is the *k*-th centroid.


(6) $$\bar{x}=\frac{\sum_{i=1}^{N}D}{N}$$

(7) $$s=\sqrt{\frac{\sum_{i=1}^{N}{\left(D_{i}-\bar{x}\right)}^{2}}{N}}$$

**Figure 4:**
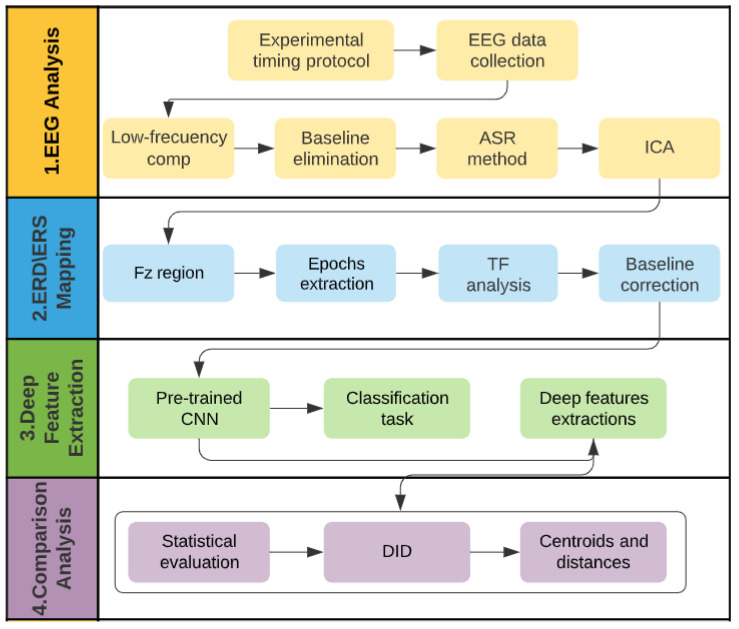
Pipeline of the EEG analysis to evaluate the effectiveness of ADT to treat subjective, chronic, and refractory tinnitus.

In summary, the pipeline of the EEG analysis undertaken for this research was followed in four stages: (1) EEG Analysis, (2) ERD/ERS Mapping, (3) Deep Feature Extraction, and (4) Comparison Analysis. Figure 4 presents in detail the whole pipeline.

## 3. Results

Table 3 shows the training and validation accuracies of the MobileNet-V2 model used in the current research study. Although the classification metric is not the main purpose of the work, the classification percentage was reported to obtain a reference of the model performance used for the extraction of deep features.

Table 4 shows the clinical (laterality, frequency, and intensity of tinnitus, heart rate, and hearing loss) and demographic (age, sex) characteristics of the study sample of tinnitus patients.

From the 11 participants, five were selected. The rest of them were rejected for any of the following two reasons: there were no auditory material recognition responses in the initial monitoring session during the acoustic therapy or during the pre-processing stage due to segment rejection for artifacts, and/or the channel Fz was eliminated due to the transient or large amplitude artifacts.

**Figure 5:**
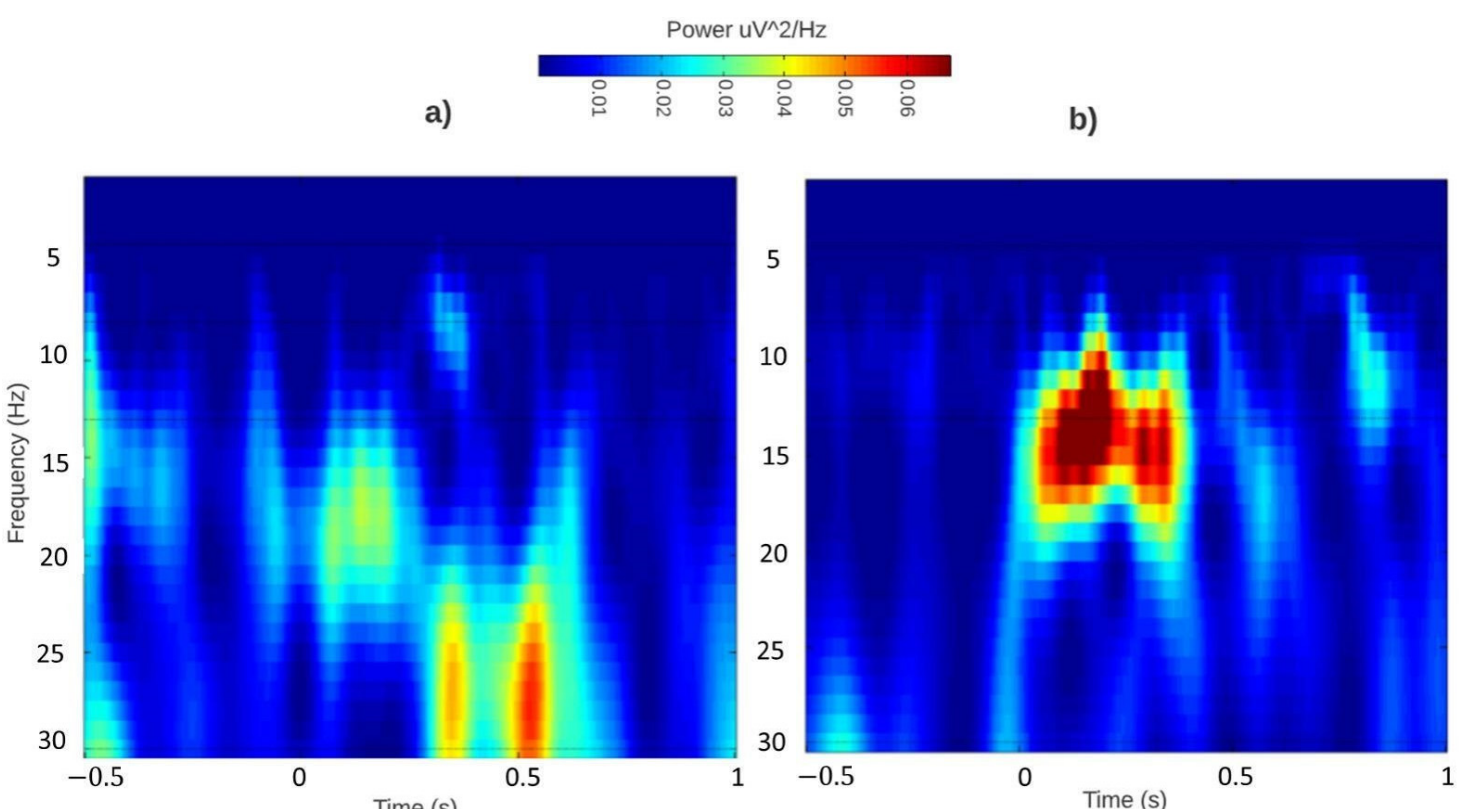
Tinnitus group. ERD/ERS responses over Fz before (**a**) and after (**b**) ADT-based treatment during the auditory recognition event. Fz was selected to illustrated central tendencies since it is the clinical recording site to diagnose tinnitus.

**Figure 6:**
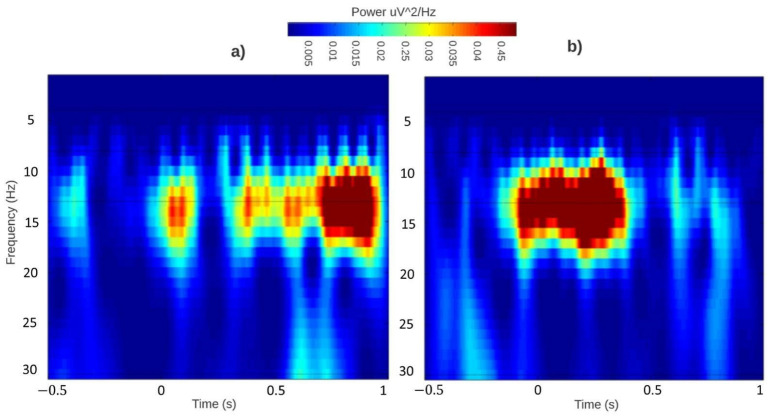
Control group. ERD/ERS responses over Fz before (**a**) and after (**b**) the sound-based treatment during the auditory recognition event. Fz was selected to illustrated central tendencies, since it is the clinical recording site to diagnose tinnitus.

Event-related (de) synchronizations maps extracted during the auditory recognition task before and after the sound-based treatment are shown in Figure 5 and Figure 6.

In Table 5, we can see *p*-values as a result of the Student’s *t*-test to statistically assess all tinnitus patients and control participants before and after the sound-based treatment under the experimental condition related to the recognition of acoustic material. Estimations indicated with a plus sign refer to those *p*-values above 0.05. These represent a statistically significant relationship in the ERD/ERS responses between the two study conditions. On the other hand, in Table 6, we can see *p*-values as a result of the Student’s *t*-test to statistically assess each tinnitus patient and all control participants before and after the sound based treatment under the experimental condition of recognition of acoustic material. Estimations indicated with a plus sign refer to those *p*-values above 0.05. These represent a statistically significant relationship in the ERD/ERS responses between the two stated study datasets.

**Figure 7:**
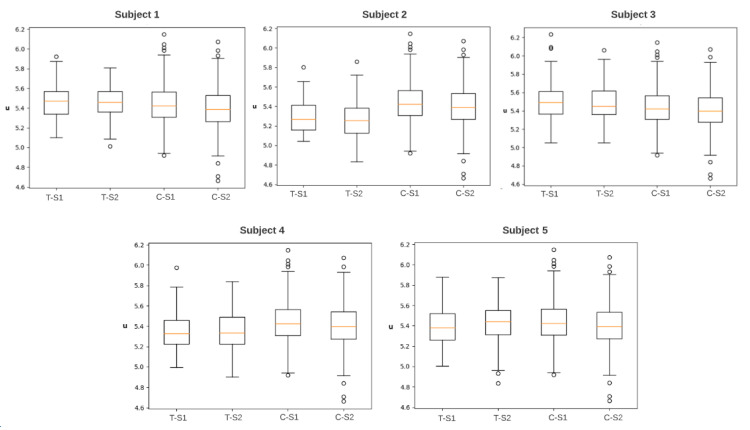
Box plots of five subjects as a result of between-subjects design to obtain the statistical distribution between-tinnitus subjects versus control participants in two monitoring sessions. T-S1: tinnitus group before the sound-based treatment, T-S2: tinnitus group after the sound-based treatment, C-S1: control group before the sound-based treatment, C-S2: control group after the sound-based treatment.

**Figure A1:**
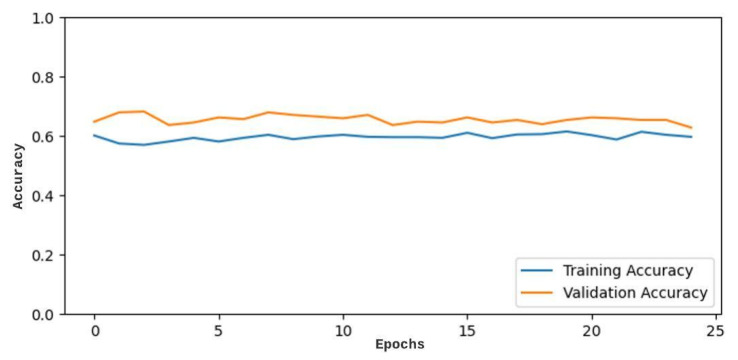
Training and validation curves to display the classification performance of the MobileNet-V2 model used in the current research study.

In Figure 7, boxplots display the distribution of the different study datasets: tinnitus and control groups in two monitoring sessions: before and after the sound-based treatment.

Table 7 shows the differential effect of the sound-based treatment on the ‘tinnitus group’ versus the ‘control group’ in both experimental designs: between-subjects and within-subjects. The DID negative refers to a negative therapy effect, whilst positive estimators have to do with a positive treatment effect.

On the other side, in Table 8, we can see the means and standard deviations of Euclidian distances between each data instance of tinnitus and control groups before and after the treatment with regard to the corresponding control centroids with the aim to measure the closeness among the different study groups.

## 4. Discussion

The aim of this study was to establish an objective methodology based on EEG analysis to measure changes in attentional processes in tinnitus patients treated with ADT.

Regarding the ERD/ERS responses of the tinnitus group (Figure 5), the absence of ERS response during the initial monitoring session (before ADT) and the increase in 4–13 Hz ERS during the final monitoring session (after ADT) could indicate increased cognitive demands such as semantic memory (cognitive processes responsible for accessing and/or bringing back information from long-term memory) and attentional processes [37] during the performance of the experimental task. Moreover, regarding [1,38], the alpha power increase in the final session may indicate that the ADT-based treatment had increased attention to everyday acoustic environments, and tinnitus sufferers were able to identify typical related auditory stimulus. Furthermore, during the first session, high-frequency energy is observed between 25 and 30 Hz after 500 ms of the stimulus onset. This could mean that tinnitus patients were able to identify the auditory stimuli at high frequencies as they perceived the task with a high complexity level because alongside the tinnitus sounds, they heard their own tinnitus causing a division in their attention. Nonetheless, during the final monitoring session, the responses are observed as normal. In addition, there was a notable decrease in the reaction time from 0 to 500 ms, and there was a frequency decrease in the neurons communication with the aim to meet the task.

On the other hand, ERD/ERS responses of the control group (Figure 6) kept high levels of synchronization within the alpha band in both monitoring sessions, which could indicate that the semantic memory was maintained throughout the sound-based therapy. However, the reaction time was changed as well. During the first monitoring session, there was a dispersed reaction time from 0 to 1 s as the experimental paradigm is new for the subjects. Therefore, the reaction times were more diverse. Even so, the central tendency is tardy, closed to the second 1. On the other side, during the final monitoring session, such variability decreases considerably downsizing the reaction time range from 1 to 500 ms.

One recurring problem with tinnitus research is that there is no objective way of assessing whether treatments counteract tinnitus. A recent systematic review examined the work to date on trying to find suitable objective measures of tinnitus [39]. The authors identified 21 articles, studying objective tests that included blood tests, electrophysiological measures, radiological measures, and balance tests. They concluded that the quality of evidence was generally poor and had failed to identify any reliable or reproducible objective measures of tinnitus. According to a subjective comparison among several acoustic therapies with the aim to evaluate the effect in tinnitus patients through a psychological evaluation [27], the retraining treatment was the most effective sound-based therapy to reduce tinnitus perception and to release stress and anxiety after 60 days of treatment. Nonetheless, binaural sounds and ADT produced very similar effects. Furthermore, ADT showed to exert less side effects. Nonetheless, nothing has yet been shown to offer the necessary specificity and sensitivity to be used as a biomarker in tinnitus treatment. As findings have shown, considerable variability and lack of consistency among studies suggest that further work in this area is needed [25]. Unlike the current research study, we herein proposed a quantitative approach based on EEG analysis and deep feature extraction to objectively measure ADT-based treatment comparing the tinnitus group with a control group to ensure reproducibility and sensibility measurement. A recent study by [28] combined objective and subjective measures to evaluate the effect of BST in tinnitus patients. The THI questionnaire reported that BST increased tinnitus perception in 15% of the patients. Furthermore, according to EEG monitoring, BST did not tend to reduce tinnitus perception but instead appeared to reduce tinnitus distress due to the slightly major neural synchronicity over the right frontal lobe found after the treatment. Unlike the current research, a new methodology was herein proposed as a first approach to evaluate the effect of the ADT-based treatment by EEG analysis.

In contrast to evoked activity, induced response refers to modulations of ongoing neural activity commonly quantified by event-related oscillations (EROs). As EROs reflect the coupling and uncoupling of neural networks, these EEG parameters give an insight into the functional neural network dynamics [5]. As far as it is known, ERD/ERS has not been undertaken to monitor electrophysiological changes in tinnitus sufferers during an acoustic therapy, it had been exemplified above the versatility of ERD/ERS estimation to capture the dynamics of neural oscillations related to emotional, cognitive, perceptual, and motor events [5]. Based on the previous statement, ERD/ERS maps were extracted so that deep features can be carried out to quantify the level of synchrony of the EEG signals by performing a cross-sectional study, comparing the tinnitus patients with control subjects at the end of the ADT-based treatment.

Based on [12], we supported the notion that tinnitus heterogeneity influences the observed variability in treatment response after an analysis of collected data of 5017 tinnitus bearers where participants reported which treatments they tried, the duration and the outcome of the given treatment, alongside with the demographic and tinnitus characteristics. Sound therapy can effectively suppress tinnitus, at least in some patients [40], but there is still a lack of research on the efficacy of sound therapy. It is necessary to analyze the characteristics of individual tinnitus patients and to unify the assessment criteria of tinnitus [24]. In Table 6 and Table 7, *p*-values above 0.05 and DID results suggest all the adult patients had a positive effect after the ADT-based treatment, whilst the elderly patient, under the same experimental conditions, had a negative effect. Furthermore, the subject who faced a significant improvement having the highest DID estimator and a similar statistical distribution to the control groups before and after the sound-based treatment is the one with the lowest tinnitus intensity registered, alongside with low hearing loss in both ears.

Regarding treatment duration, it should be interpreted with caution, as it is well-known that certain treatments require some time for adaptation, whereas other treatments require longer periods to be effective [12]. There is still uncertainty about the duration of treatment that may be required to achieve an improvement [25]. During this study, ADT-based treatments lasted 8 weeks. However, they were not applied for all patients even though 2 months is the minimum necessary time that has been empirically reported to find changes [12].

Tinnitus impairment can be quantified by various validated questionnaires such as THI. However, a recent analysis revealed a high variability in the outcome instruments used in clinical trials, indicating the need to standardize outcome measurement [9]. Furthermore, the outcome measures carried out through the THI in [12] were retrospective and subjective, which could have biased the results. This is why questionnaires are considered a subjective metric. According to [25], a further limitation of the current tools for assessing tinnitus impact is the reliability and repeatability of such measures: self-report measures of tinnitus have an associated risk of variability, as they supply a momentary snapshot, whereas the experience of tinnitus changes with time and context. Based on the previous evidence, it was proposed a first quantitative approach to objectively measure and evaluate the effects of ADT using ERD/ERS techniques along with the extraction of deep spectrum features. Significant relationship responses between the ‘tinnitus group’ after the sound-based treatment versus the ‘control group’ (Table 5 and Table 6), positive DID estimators (Table 7), and close distance measures (Table 8) indicate the existence of neural modifications, which could explain why this treatment is so effective in some scenarios. Results from this research might help point out ADT as a potential solution for certain patients, but it is not a viable treatment for many others.

According to [24], patients with more severe initial tinnitus respond better to sound therapy; however, in the current study, the opposite results were observed. In Table 6 and Table 7, *p*-values above 0.05 and positive DID estimators suggest that the subject who faced a better performance is the one with the lowest tinnitus intensity registered, alongside with low hearing loss in both ears. The elderly patient who did not benefit from acoustic therapy was due to the time he had suffered from tinnitus: around 30 years.

Our study comes with some inherent limitations. First, although we started analyzing 11 tinnitus patients, this number was reduced to 5 tinnitus subjects due to one of the following reasons: the rest did not show auditory material recognition responses in the initial monitoring session before receiving the ADT-based treatment or during the preprocessing stage, and the channel Fz was eliminated due to the transient or large amplitude artifacts. The final sample was insufficient, so it might not be representative of all patients with tinnitus. Second, the improvement trend is inevitable; however, it would be interesting to carry out a deep spectrum features analysis by theta, alpha, and beta bands to know exactly which cognitive demands are increasing or decreasing in terms of semantic, working memory, and attentional processes in each tinnitus subject compared with control subjects.

## 5. Conclusions

In conclusion, a new methodology based on ERD/ERS analysis and deep spectrum features extraction was successfully implemented to measure changes in attentional processes in tinnitus patients treated with ADT. Based on the previous implementation, our results pointed out that tinnitus attention was significantly reduced after the ninth week of an ADT-based treatment in adult patients. Furthermore, the therapy reported significant improvements in the patients with the lowest intensity recorded of tinnitus, alongside with low hearing loss in both ears. It is worth mentioning that this acoustic therapy is based on redirecting the attention that the patient has his tinnitus, this attention is focused on the deviant pulse of the oddball paradigm that is different from the frequency of the tinnitus. After eight weeks of treatment, the patient reports a reduction in the perception, but beyond the reduction in the level of tinnitus perception, there is a reduction in the attention level, which results in the improvement of the patient.

Future work will entail measuring the EEG signals over the whole frontal lobe (Fp1, Fp2, F7, F3, Fz, F4, and F8). Furthermore, different neural network architectures could be applied to ensure the increase of the accuracy percentage in the classification stage to make the deep feature extraction stage more reliable.

## Figures and Tables

**Table 1:** EEG recording system configuration.

Sampling rate	256 Hz
Number of channels	16
Channels used by region	Prefrontal (FP1, FP2), Frontal (F7, F3, Fz, F4, F8), Temporal (T3, T4, T5, T6), Central (C3, C4), Parietal (Pz), Occipital (O1, O2)
Reference method	Monopolar @ Cz
Electrode placement system	International 10–20 system

**Table 2:** Study groups. Tinnitus vs. Control group.

			Tinnitus			
			Intra-Subject Comparison		Inter-Subject Comparison	
			Before	After	Before	After
Control	Intra-subject comparison	Before	X	X	X	X
		After	X	X	X	X

**Table 3:** Training and validation accuracies of the MobileNet-V2 model used in the current research study.

Epochs	Training Accuracy	Validation Accuracy
1	0.6321	0.642
2	0.6324	0.6307
3	0.6331	0.625
4	0.6341	0.6364
5	0.6345	0.6335
6	0.635	0.6349
7	0.637	0.6359
8	0.6477	0.6392
9	0.6511	0.6449
10	0.6623	0.6492
11	0.681	0.6392
12	0.682	0.644
13	0.6874	0.6392
14	0.681	0.6477
15	0.682	0.6591
16	0.681	0.66
17	0.6825	0.672
18	0.6835	0.6899
19	0.6855	0.6899
20	0.6817	0.6909
21	0.682	0.6591
22	0.681	0.66
23	0.6825	0.672
24	0.6835	0.6821
25	0.6855	0.6899
Average	0.6758	0.661684211

**Table 4:** Clinical and demographic characteristics of the study sample: Tinnitus patients.

Subjects	Age	Sex *	Laterality **	Frequency [Hz]	Intensity [dB]	BPM ***	HL ****-L	HL-R
1	Adult	M	R	125	90	75	96	20
2	Elderly	M	R	6000	70	79	56	52
3	Adult	M	L	8000	50	69	29	30
4	Adult	F	B	2000	87.5	86	63	70
5	Adult	F	B	6000	20	*	13	10

* M: male, F: female, ** R: right, L: left, B: both. *** BPM: beats per minute. **** HL: hearing loss → L: left and R: right.

**Table 5:** *p*-values as a result of within-subjects design where the Student’s *t*-test was applied in tinnitus subjects versus control participants in different sessions undertaken before and after the sound-based treatment.

	Tinnitus S1 *–Control S1	Tinnitus S1–Control S2 **	Tinnitus S2–Control S1	Tinnitus S2–Control S2	Tinnitus S1–Tinnitus S2	Control S1–Control S2
Tinnitus Patients	-	-	+	-	+	
Control Patients						-

* S1: before the sound-based treatment. ** S2: after the sound-based treatment. -: significant differences (*p* < 0.05). +: significant relationship (*p* > 0.05).

**Table 6:** *p*-values as a result of between-subjects design where the Student’s *t*-test was applied in each tinnitus subject versus the control participants in different sessions undertaken before and after the sound-based treatment.

Tinnitus Patients	Tinnitus S1 *–Control S1	Tinnitus S1–Control S2 **	Tinnitus S2–Control S1	Tinnitus S2–Control S2	Tinnitus S1–Tinnitus S2
1	+	-	+	-	+
2	-	-	-	-	+
3	-	-	+	-	+
4	-	-	-	-	+
5	-	+	+	+	+

* S1: before the sound-based treatment. ** S2: after the sound-based treatment. -: significant differences (*p* < 0.05). +: significant relationship (*p* > 0.05).

**Table 7:** DID between-subjects and within-subjects.

Subjects	DID	ADT-Based Treatment Effect
1	0.0327	Positive effect
2	−0.0018	Negative effect
3	0.0152	Positive effect
4	0.0464	Positive effect
5	0.0741	Positive effect
All tinnitus patients	0.0225	Positive effect

**Table 8:** Distance measures among data instances of control and tinnitus groups and control centroids.

Instances-Centroids		Subject 1	Subject 2	Subject 3	Subject 4	Subject 5
Tinnitus S1 *— Control S1 **	Mean	2.0867	1.9623	1.9567	1.8840	1.9407
	STD	0.2664	0.2350	0.2798	0.2002	0.2553
Tinnitus S2— Control S1	Mean	2.1166	1.9433	1.9745	1.8803	1.9214
	STD	0.2747	0.2520	0.2733	0.2349	0.2537
Tinnitus S1— Control S2	Mean	2.0166	1.9092	2.0065	1.8570	1.9025
	STD	0.2738	0.2379	0.2830	0.2081	0.2623
Tinnitus S2— Control S2	Mean	2.0432	1.8968	2.0307	1.8560	1.8884
	STD	0.2821	0.2640	0.2852	0.2404	0.2568
Control S1— Control S2	Mean	1.7758	1.7562	1.8997	1.7025	1.8513
	STD	0.2827	0.2345	0.2969	0.2298	0.3289

* S1: before the sound-based treatment. ** S2: after the sound-based treatment.

## Data Availability

The database used in this study is available at https://data.mendeley.com/datasets/kj443jc4yc/1 (accessed on 20 November 2021).

## Appendix A

**Table A1:** Rejected channels, the percentage of bad data periods with transient or large-amplitude artifacts, and the independent components distinguished as non-brain sources.

Subjects	Sessions	Channels Rejected	Percentage of Bad Data Periods	Components Flagged for Rejection
1	1	0	17.5%	3
	2	1	0.4%	3
2	1	2	0.0%	2
	2	1	0.0%	7
3	1	2	0.0%	1
	2	2	0.0%	0
4	1	4	46.5%	4
	2	1	20.0%	3
5	1	2	13.0%	4
	2	4	15.1%	3
6	1	0	3.4%	3
	2	2	1.3%	3
7	1	12	1.9%	0
	2	3	1.4%	2
8	1	4	12.4%	2
	2	3	1.7%	4
9	1	1	5.3%	3
	2	0	0.7%	2
10	1	0	1.1%	2
	2	0	0.7%	3
11	1	1	0.7%	3
	2	4	1.1%	2
